# How and why do community stakeholders participate in the national stroke audit in England? Findings from a mixed-method online survey

**DOI:** 10.1186/s12913-024-11653-1

**Published:** 2024-11-06

**Authors:** L Russell, N Chouliara, S Lewis, M James, R Fisher

**Affiliations:** 1 University of Nottingham, Nottingham, UK; 2 NIHR Applied Research Collaboration (ARC) East Midlands, Nottingham, UK; 3 Royal Devon and Exeter NHS Foundation Trust, Devon, UK; 4 Stroke Programme, King’s College, London, UK

**Keywords:** National clinical audit, Quality improvement, Stroke rehabilitation

## Abstract

**Background:**

National audit programmes are a recognised means of assessing quality of healthcare by collecting and reporting data in relation to evidence-based standards. The Sentinel Stroke National Audit Programme is a prospective audit of processes and outcomes for all stroke patients in England, Wales and Northern Ireland which has historically focused on hospital-based care. Evidence suggests it has been successful in driving quality improvement. What has yet to be explored is the influence of such a national audit programme on community-based healthcare. The aims of this study were to understand how community stakeholders perceive and participate in the audit.

**Methods:**

The study used a realist approach, being theory driven and informed by collaborators including stroke clinicians and experts in realist and audit methodology. Contextual determinants and mechanisms were identified from the literature as having the potential to influence quality improvement. These were operationalised into 18 survey items, using a combination of 5-point scales and yes / no responses. Free text options offered the opportunity to expand upon responses.

The online survey was distributed using social media, clinical networks and professional bodies. Representation was sought from community stroke stakeholders across England and from roles throughout the audit process including administrative, clinical, management and commissioning.

**Results:**

The survey achieved a national sample from a broad range of stakeholders (n=206). Participants reported being engaged in the audit, committing significant resources to participation. National audit feedback was described as being used to support a range of improvement activities, including funding for additional staff and service reorganisation. A number of factors influenced the ability of teams to participate in audit and utilise feedback for quality improvement. These included the online platform, the accuracy of data submitted and leadership support.

**Conclusions:**

Findings highlight the work needed in terms of the data captured, organisational audit support and engagement with feedback if the potential of the audit as a tool for quality improvement in community rehabilitation (as highlighted in acute stroke care) is to be realised.

**Supplementary Information:**

The online version contains supplementary material available at 10.1186/s12913-024-11653-1.

## Background

National registries or audit programmes are a recognised means of assessing quality of healthcare delivery and driving healthcare improvements [1]. They collect a variety of information such as patient characteristics, outcome measures and provider performance measures such as length of hospital stay [2]. Internationally, a number of studies have found audit to be an effective tool for improving the quality of stroke care [3]. There are a number of established stroke specific national quality registries and audit programmes. Examples include the Scottish Stroke Care Audit [4], the Swedish Riksstroke registry [5] and the Australian Stroke Clinical Registry [6]. Differences exist between these, for example whether they are mandated and what data they collect [7].

In the UK, the Sentinel Stroke National Audit Programme (SSNAP), has been informed by the development of evidence-based national clinical guidelines [8, 9]. Like many national audit programmes, SSNAP has historically focused on acute and hospital-based care [10]. However, in line with a move over the last decade by the National Health Service (NHS) to develop community-based healthcare as prioritised in the NHS Long Term Plan, audit programmes such as SSNAP have expanded to cover post-acute or community pathways [11].

Since 2013 SSNAP have collected prospective data for all stroke patients in England, Wales and Northern Ireland [12]. This database has offered opportunities to gain insights into the quality of services delivered, such as the impact of staffing patterns and temporal variations in quality across the week [12–14]. Evidence suggests that SSNAP has been successful in driving improvements in hospital-based stroke care by highlighting inconsistencies in clinical practice or service delivery between NHS trusts, and comparison with accepted national clinical guidelines [8]. Data from the audit has informed policy initiatives within the NHS such as a national stroke strategy and the introduction of financial incentives linked to performance [12].

However, the role and impact of national audit in quality improvement in the community setting have yet to be established. In SSNAP, data is collected by clinical teams and inputted to the online platform. The process by which data is collected varies in the community but is commonly undertaken by clinical staff such as rehab support workers.

There are challenges associated with collecting national data beyond hospital-based care. In contrast with acute services which are more standardised, community stroke services are provided in the patient’s own home and over an extended period of time. Variety exists in the services commissioned, eligible patient cohorts and models of delivery [15]. This raises questions as to how to best capture multidisciplinary team (MDT) activity with a dispersed delivery of rehabilitation, and ultimately if this relates to patient outcomes [16].

Due to a lack of literature regarding audit in community MDT’s, it is also unclear how stakeholders working in community stroke care perceive audit or whether it’s used for quality improvement in this setting. For the purpose of this study, stakeholders are defined as anyone working in, leading or commissioning community stroke rehabilitation services that contribute to SSNAP. The activities that stakeholders are involved with varies based on their role and may differ between teams. For example, data is often collected by Administrators or Rehab Support Workers. Feedback reports are publicly available and may be reviewed by anyone.

The aims of this study were to understand how community stakeholders perceive and participate in SSNAP.

## Methods

### Methodological framework

This study adopted a realist methodological approach. Realist evaluations (RE) are appropriate for the evaluation of complex interventions such as audit [17, 18] and have established quality criteria which have informed this study [19]. RE seeks to develop, refine and test programme theories that explain “what works, for whom, under what circumstances and how?” [19]. In this study, the mechanisms and contextual factors that influence stakeholder engagement, participation and use of audit data for quality improvement have been explored. Programme theories consist of context, mechanism and outcome (CMO) configurations. These form a hypothesis regarding how a specific contextual feature, or combinations of features may influence the outcomes of interest, via an underlying mechanism [18]. Context describes the conditions in which an intervention occurs and determines the degree to which a mechanism is triggered, if at all [20]. Definitions of mechanisms vary, for the purposes of this study mechanisms are defined as the interaction between the resources offered by the intervention (in this case SSNAP) and stakeholder reasoning in responses [21]. Outcomes of an intervention result from the activation of mechanisms within a context and may be intended or unintended [22].

Collaborator engagement is fundamental to RE. It is recommended that researchers gather a group of “experts” to regularly sense-check, inform the focus of enquiry and validate emergent findings [23, 24]. This study involved 11 collaborators, with expertise in stroke rehabilitation (*n* = 5), audit methodology (*n* = 3) and realist methodology (*n* = 3). Meetings were completed virtually, either individually or in groups of two or three.

The proposed initial programme theories (IPT) were informed by a preliminary scoping review of the literature. This focussed on literature reporting multidisciplinary clinical audits in high- and middle-income countries, with a desired outcome of quality improvement. Contexts were identified from the literature in which audit was reported to contribute to quality improvement, or the mechanisms by which this was reported to occur. Although studies identified were predominantly hospital or clinic based, findings provided a platform from which to explore the community context. These were prioritised and nuanced to the setting of community stroke rehabilitation through collaborator discussions. The resultant proposed IPT’s were used as a framework for exploration, an overview is provided in Table 1.

**Table 1:** Overview of proposed initial programme theories

Proposed IPT	Context	Mechanism	Outcome
1. Individual perception of audit influences motivation to engage	If individuals perceive audit to be a worthwhile activity	Then they are motivated by the potential benefits	Individuals will engage with the audit
2. If information regarding the audit is available, individuals are empowered to participate	If the purpose and process of audit is explained and roles articulated	Then individuals understand the audit and have insight what is expected of them. Consequently, they are empowered to participate	Individuals will complete the audit tasks appropriate for their role
3. If stakeholders have resources to support participation, data will be inputted completely and reflect the caseload	If resources such as computers are available to complete audit activities	Then individuals are enabled by the resources and motivated by the perceived value placed on the audit by their organisation	Data inputted will be complete for the caseload
4. If data is perceived as accurate then it will be used to inform quality improvement	If data contained in feedback report is perceived as accurate	Then the report will be perceived as trustworthy, and individuals will have the confidence to act upon it	Audit feedback is used to inform quality improvement

Consistent with realist approaches, this study used a mixed methods, explanatory design to explore theories in greater depth [25]. An online survey was chosen to access a national sample of stakeholders. This captured the perspectives of a broad range of individuals in different roles, regarding their experiences of the audit. The survey collected predominantly quantitative data regarding context and outcomes. Free text responses offered opportunities for elucidation or expansion. The GRAMMS Framework for reporting has been adhered to [26]. Narrative integration occurred at the interpretation and reporting level. A weaving approach has been used, whereby quantitative and qualitative findings for each proposed IPT have been presented together [27]. The intention of this mixed methods approach was to generate a more complete understanding than would be possible from quantitative or qualitative findings alone [28].

### Survey design

The survey was designed and reported in line with the Checklist for Reporting Results of Internet E-Surveys (CHERRIES) [29] (Supplementary File 1). The survey was developed in three stages: content, logic and finally piloting and refinement.

#### Content

Attempts were made to articulate components of the proposed context, mechanism and outcome for each proposed IPT as survey items. Feedback from collaborators informed the choice of language, format and underpinning conceptualisation of each item. Not all components could be articulated as survey items. For example, mechanisms may explore behaviours which are difficult to quantify, therefore free text options were used to explore and capture these where possible. This resulted in 18 survey items (Supplementary File 2). An example is included in the Table 2 below.

**Table 2:** Example of context, mechanism and outcome (CMO) articulation

CMO Configuration	Articulated as survey item
**Context** The purpose and process of audit is explained, and roles / expectations articulated	-I understand the purpose of the audit -I understand what my role is in the audit -I understand what activities I need to complete for the audit -I understand how to complete the required activities (5-point Likert response options)
**Mechanism** Individuals have an understanding of audit and insight what is expected of them which empowers them to participate	-If you are unable to fully complete the audit tasks required for your role, please explain why (Free text response)
**Outcome** Stakeholders will complete audit tasks appropriate for their role	-Indicate from list which audit activities you participated in (Options include “other”) -Are you able to fully complete audit activities required for your role? (Yes or no response options)

#### Logic

All survey items were mandated. A combination of categorical, free text, yes / no and five-point Likert scale response formats were utilised. Free text options were included for expansion upon yes / no answers and offering examples where “*Other*” was selected from a list of categorical response options. Likert scales were utilised to explore participant perceptions, establishing their agreement with a number of statements. Response options included “*agree completely”*,* “agree partially”*,* “neither agree or disagree”*,* “disagree partially”* and *“disagree completely”.* The use of named categories such as these has been found to provide acceptable levels of reliability and be user-friendly [30].

#### Piloting and refinement

The survey was piloted, using different audiences for specific purposes. Collaborators provided feedback regarding the logic, coherence and functional utility of the tool. Clinical colleagues and collaborators with audit experience provided feedback on the clarity and technical content.

### Sampling and data collection

Between 01.12.2021 and 01.04.2022, an advert was circulated via social media and professional networks. Individuals who worked in, managed or commissioned a community stroke rehabilitation team collecting SSNAP data were invited to participate. Online surveys require basic digital literacy and access to a device such as a computer or android telephone [31] which would be achievable for potential participants. Study information and contact details were available on the first page of the survey, followed by participant consent, which was mandatory for participation. To gain causal insights from a variety of stakeholders, efforts were made using established clinical networks to disseminate the advert widely. Consistent with RE, representation was sought from diverse stakeholders in terms of role and geographical region. Participant numbers were expected to vary between categories, reflecting the number of individuals in these roles e.g. there are more clinicians employed within community stroke than commissioners. This is commensurate with RE as the purpose of sampling is to illuminate different facets of the intervention [32] rather than seek statistical significance.

Participants accessed the survey via a secure link in the advert. The online platform (Jisc Online surveys™) stored participant responses. Once the survey had closed, quantitative data were exported to a Microsoft Excel™ file and qualitative data were exported to Nvivo™ software for organisation and analysis.

### Data analysis

For quantitative data, descriptive statistics were used to illustrate participant responses. For the purpose of the narrative reporting of Likert scales, agreement was defined as an aggregation of “*agree completely” and “agree partially”* responses. Following this, analysis of qualitative data from free text responses followed an iterative process of realist theory refinement as proposed by Dalkin et al. [33]. Although undertaken primarily by a single researcher (LR) to enhance rigour, excerpts of raw data, early coding, integration and theory refinement were discussed with collaborators. Both quantitative and qualitative data were exported into Nvivo™ software which supported the following process:

### IPT development

A single category was created for each IPT with an associated text document. Any refinements made to the proposed IPTs were tracked within the text document. Where causal insights were identified that did not fit into existing categories, additional ones were created.

### Coding

Quantitative data was coded to relevant categories. Qualitative data was coded to relevant categories using export coding. This approach extracts direct sections of text and was used due to the often succinct free text responses provided [34]. Consistent with realist methodology, both an inductive and deductive approach to analysis was taken [19]. The deductive framework was provided by the proposed IPT’s whilst analysis was open to new inductive insights from the data.

### Proposed IPT refinement

Refinement occurred in the presence of sufficient data to challenge or expand upon theories and was tracked using the text document as described. Where insufficient data existed to support or challenge components of a theory, this was identified as “unsubstantiated” [33]. The resulting theories were collated and further refined in light of any similarities or overlaps identified [35]. Final refinements were made with input from collaborators who offered critical reflection on the articulation, clarity and logic of theories. This process started with four proposed IPTS, which have been expanded upon and refined to the context of community stroke rehabilitation. For IPT3 the proposed context-mechanism was unsupported therefore survey findings were used to identify an alternative proposition.

## Results

A total of 206 participants completed the survey. Table 3 illustrates the breakdown by participant role. Representation was achieved from across the seven regions of England.

**Table 3:** Participants by category of role

Role	Abbreviation	n
Administrative Support	Admin	22
Rehabilitation Support Worker	RSW	19
MDT member > Band 5	MDT	53
Team Lead (clinical)	Team Lead	53
Team Lead (non-clinical)	Team Lead-NC	24
Service Manager	Manager-S	19
General / Divisional Manager	Manager-G	9
Commissioning	Commissioner	7

The following sections are organised around the four proposed IPTs in turn. Findings related to each proposed IPT are summarised. C, M and O in brackets are used to indicate findings related to context, mechanisms or outcomes that have informed theory refinement. Finally the CMO configurations for each refined IPT is presented figuratively.

### Proposed IPT-1: An individual’s perception of audit influences their engagement

**Fig. 1:**
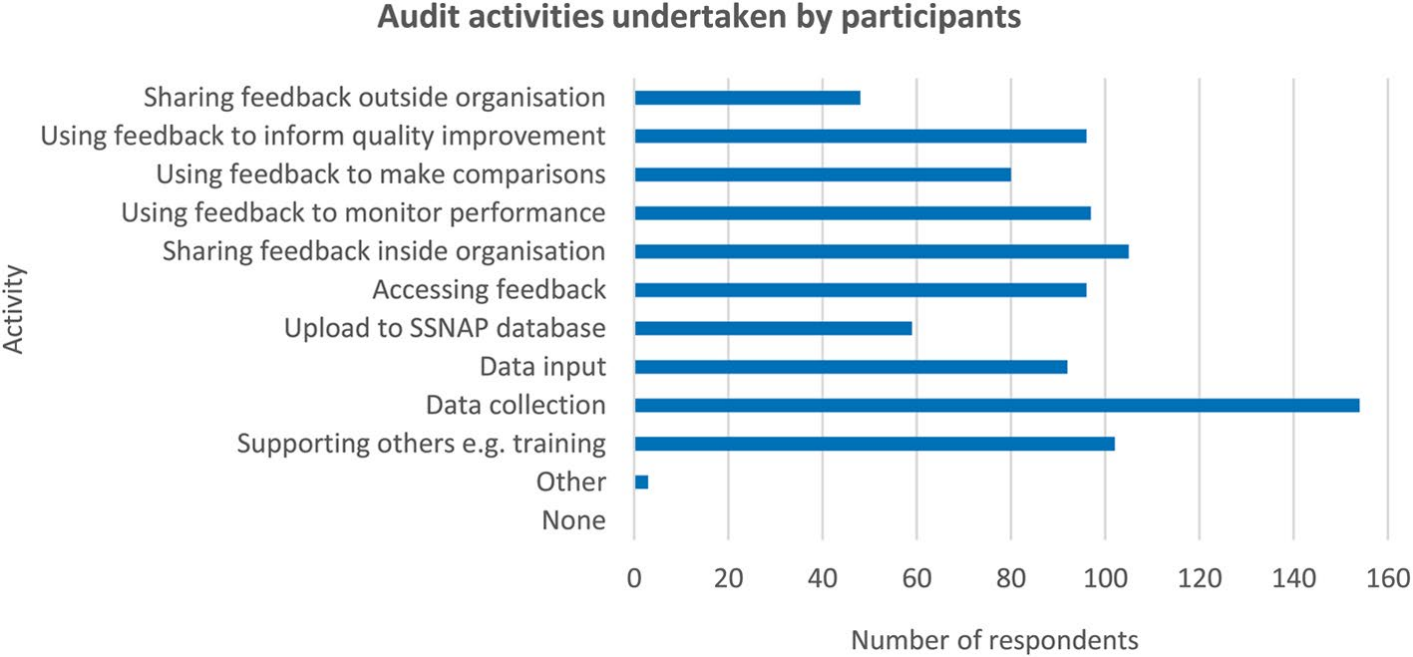
Graph illustrating audit activities undertaken by participants

When asked, 60% of participants agreed that participating in the audit was a worthwhile use of their time **(C)**. All participants reported engaging in at least one audit activity as part of their role **(O)**, the distribution of responses is illustrated in Fig. 1. When asked about the perceived benefits of the audit, 58% agreed it benefitted their service and 55% agreed it benefitted their patients. 86% of participants accessed resources to support their engagement in audit e.g. SSNAP webinars or newsletters **(M)**.

Qualitative data identified contextual features that influenced perceptions of the audit being a worthwhile activity **(C)**. These included experiences of feedback being used critically *“It can feel like the data and report is a stick to beat us by not an enabler for conversations and improvement.”* (P26:Team Lead), as well not experiencing change following engagement with audit.

“Clinicians need to see positive change …it’s no point collecting data and not taking it forward for service improvement. Otherwise, you do not get buy in.” (P3:Team Lead).

Participants reported they experienced a lack of support to act on audit findings, both leadership and financial **(C)**. The perceived lack of leadership support to act on audit findings in the community was described as resulting from “*organisational priorities lying elsewhere* [acute services]”(P153:MDT) **(C)**. Participants described these experiences as reducing motivation towards **(M)**, and ultimately reducing engagement with audit activities **(O)**. *“It’s difficult to get motivated or motivate the team if we know there is no funding to make changes.”* (P178:Team Lead).

**Fig. 2:**
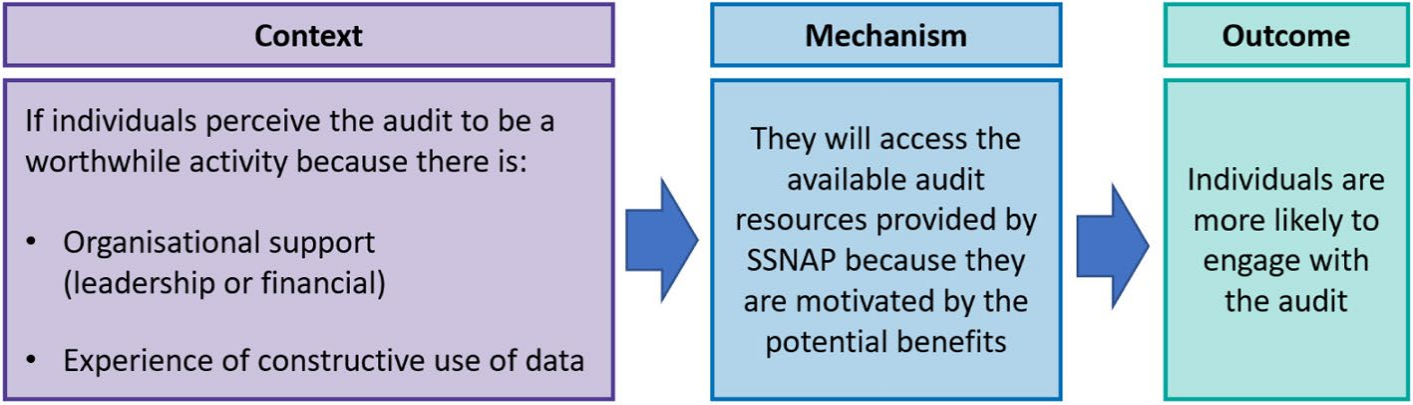
Refined IPT1 – Perceptions of audit influence engagement

These qualitative findings supported the refinement of the proposed context, offering a deeper understanding of the contextual features that contributed to a perception of the audit being a worthwhile activity. The refined IPT1 is illustrated in Fig. 2.

### Proposed IPT-2: If information regarding audit is available, individuals are enabled to participate

**Fig. 3:**
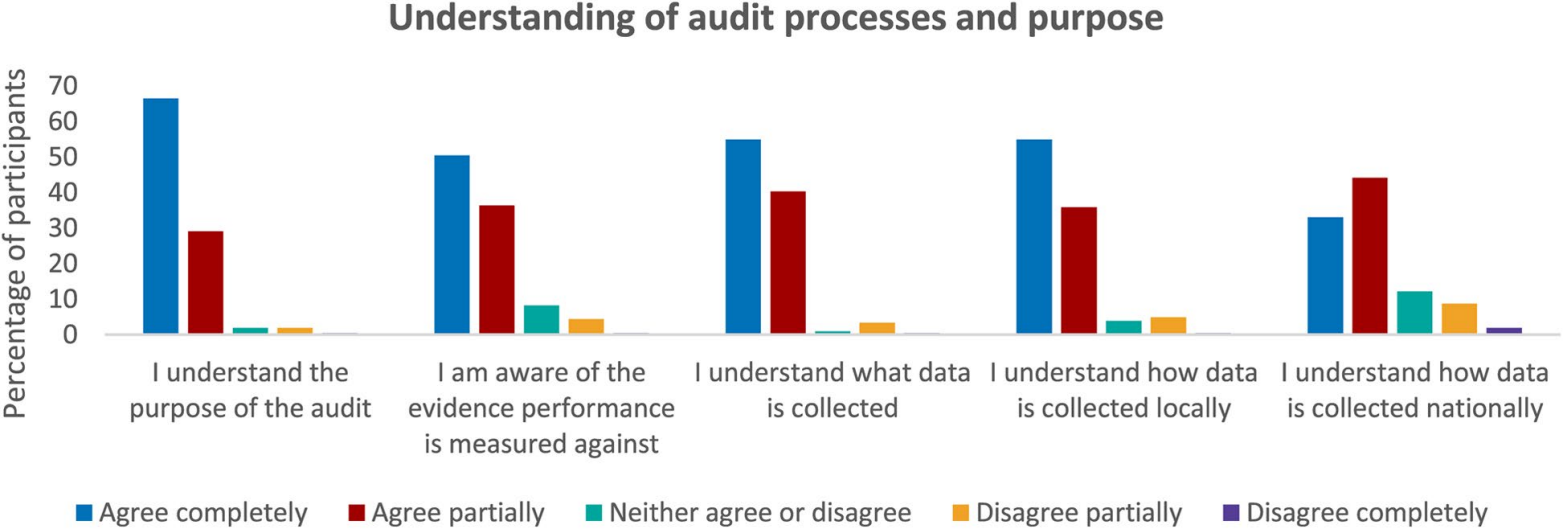
Graphs illustrating participant understanding of audit processes and purpose

As highlighted earlier, 86% of participants reported they accessed resources to support their engagement in audit such as webinars or guidance documents **(C)**. The majority of participants agreed they understood the purpose of the audit and the processes involved **(M)** as illustrated in Fig. 3.

**Fig. 4:**
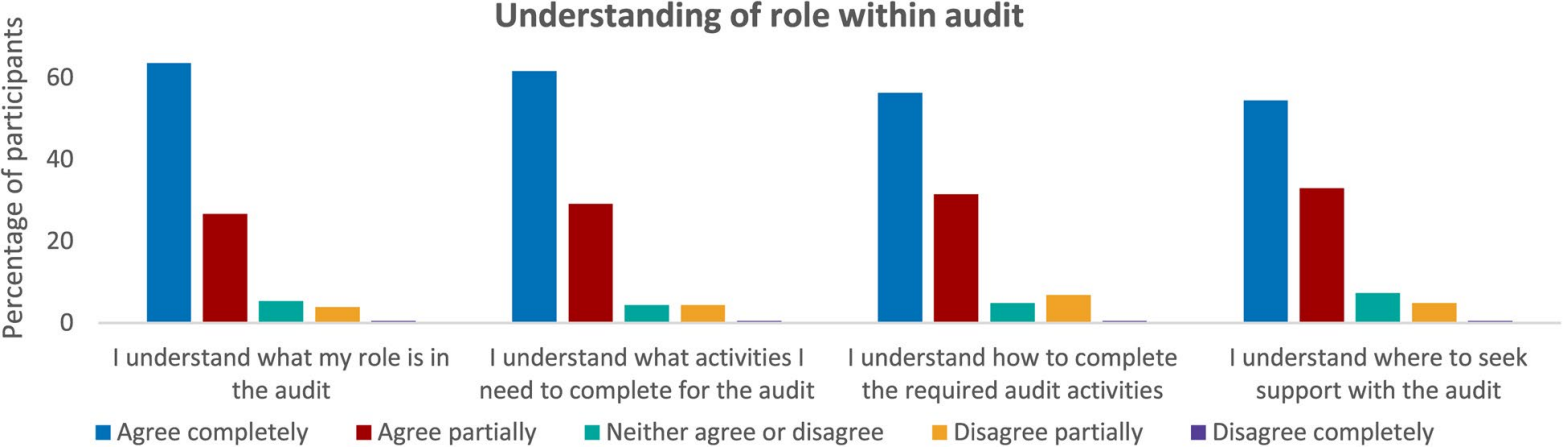
Graphs illustrating participant understanding of role within audit

Similarly, the majority of participants agreed they understood their role in the audit **(M)**, Fig. 4.

Despite participants reporting insight into the audit, only 31% of participants agreed they were able to participate fully and complete the activities required for their role **(O)**. This suggested an additional contextual feature influenced participation in audit.

For those who reported being unable to complete audit activities, time was the most commonly cited barrier in free text responses. This was most commonly reported for those with combined roles such as clinical and administrative **(C).** Less than half (48%) of participants reported they were able to prioritise audit tasks against competing demands **(M)**. *“When I’m busy patient discharge takes priority.”* (P180:RSW) Participants with responsibilities for multiple services also described a lack of dedicated time for audit **(C)** as resulting in challenges prioritising audit activities **(M)**. *“This is one of many areas I am responsible for I can’t always ring-fence time.”* (P196:Commissioner) Audit was described as an additional activity to complete, rather than an acknowledged part of a core role. *“SSNAP is not a recognised (time given) part of my role*,* therefore it is in addition.”* (P32:Team Lead-NC).

**Fig. 5:**
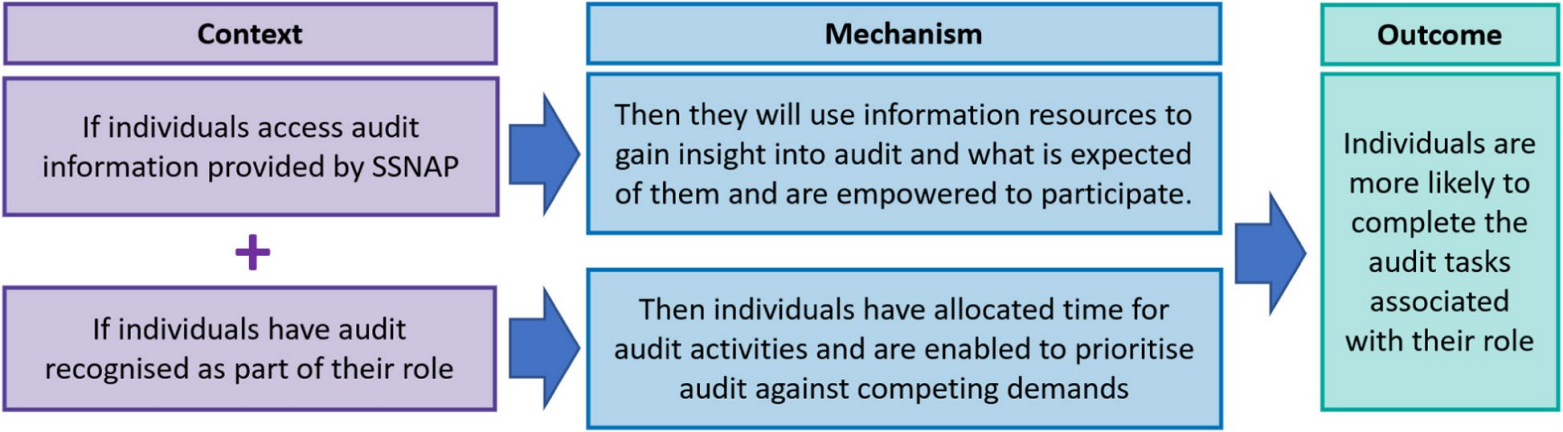
Refined IPT2 – Participation influenced by available information and audit being recognised within role

Findings support the proposed context-mechanism configuration whereby individuals gain insight into the audit as a result of accessing provider information. However, without audit being an acknowledged part of their role, individuals described challenges to participation. Therefore, recognising audit as part of an individual’s role been added as an additional contextual feature than enables participation. The refined IPT2 is illustrated in Fig. 5.

### Proposed IPT-3: If equipment is available to support participation, data will be inputted completely and reflect the caseload

The majority of participants (91%) reported they had the equipment necessary to support their participation in the audit e.g. computers / tablet devices **(C)**. Despite this, just over half (54%) reported data to be complete for all stroke patients seen by their service **(O)**. Free text responses suggested that rather than the availability of physical resources such as computers, a context of challenges with the online platform were responsible for data being incomplete **(C)**.

**Fig. 6:**
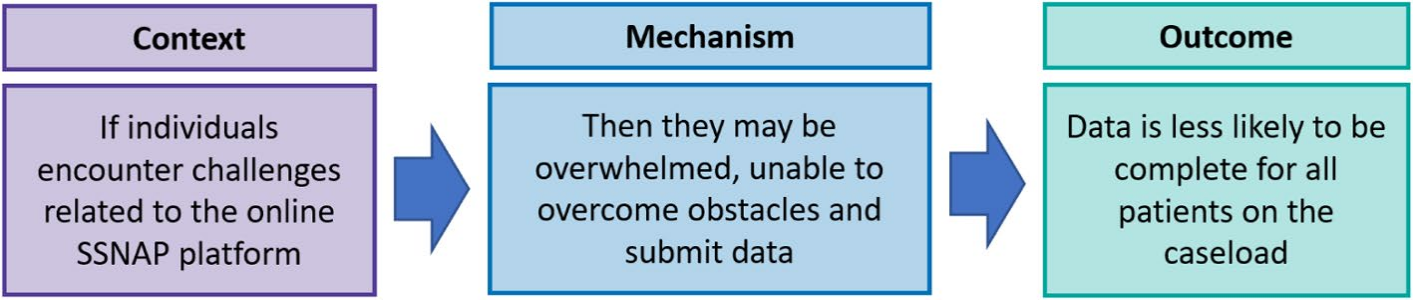
Refined IPT3 – Challenges regarding the online platform influence the submission of complete data

Participants described challenges such as “*incompatible IT systems*” (P59:Admin) **(C)**. A dependence on others to complete and lock records on the online platform **(C)**, which required “*a huge amount of time chasing the acute teams to input their data.*” (P204:Admin) Findings suggested the challenges posed by the platform **(C)** can overwhelm individuals **(M)**, impeding their ability to submit complete data for all patients **(O)**. *“Transferring records is such an arduous task…some patients just never get done.”* (P54:Admin) The refined IPT 3 is illustrated in Fig. 6.

### Proposed IPT-4: If data is perceived as accurate then it will be used to inform quality improvement

**Fig. 7:**
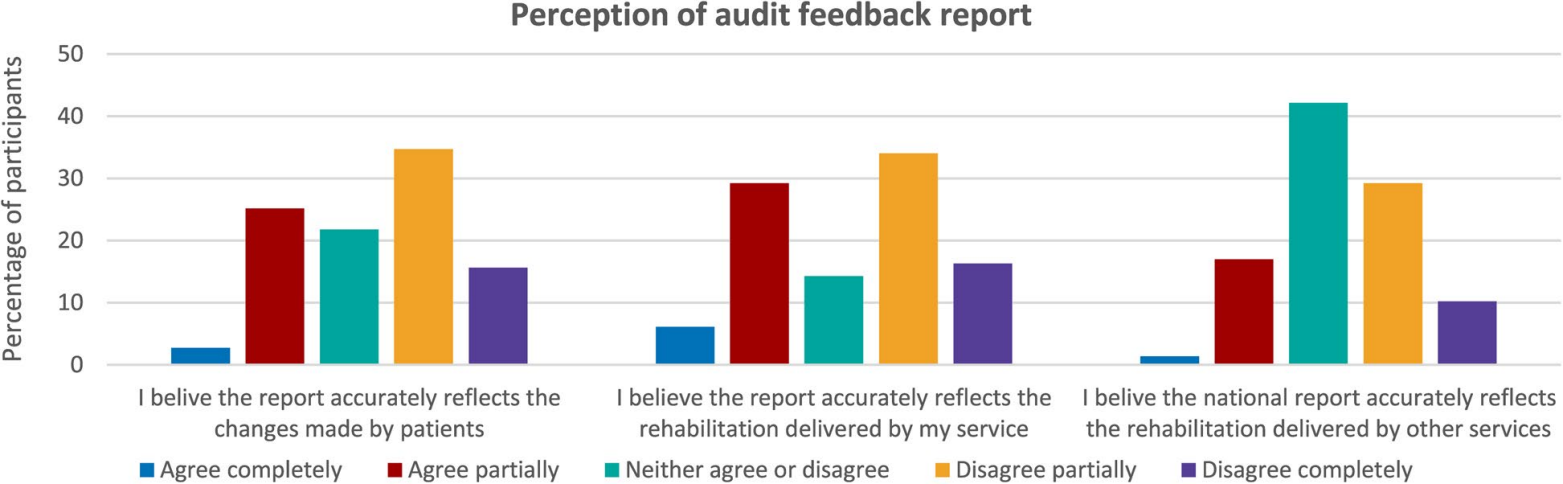
Graphs illustrating respondent perception of audit feedback report

The audit feedback report consists of summative data and a portfolio of key performance indicators for teams that submit sufficient data. 71% of participants reported they accessed this feedback. When asked, 28% of those with access to audit feedback perceived it accurately reflected the recovery made by patients and 35% agreed the report accurately reflected the service they delivered **(C)**. Only 18% of participants perceived the report accurately reflected the service delivered by other teams **(C)** see Fig. 7.

Concerns regarding the accuracy of audit feedback were expanded upon in free text responses **(C)**. These reservations were reported as reducing confidence to act upon the report **(M)**. Participants reported the data “*fails to capture the entirety of a service”* as a result of limiting data collection to six-months **(C)** (P13:Team Lead). In contrast to acute care which was perceived as more accurately captured by the audit, community feedback was described as *“failing to reflect the myriad of community commissioning models.*” This was reported as resulting in an inability to accurately reflect activity outside traditional models of rehabilitation **(C)** (P112:Manager-S).

Of those participants with access to feedback, 44% agreed the report was trustworthy. Participants described their confidence in data accuracy as undermined **(C)** by mistrust regarding the reporting practices of other teams **(M)**. Concerns were raised regarding *“huge discrepancies between teams in how data is recorded*,* reported and interpreted”***(C)** (P37:Team Lead). Perceived discrepancies were described as making it *“difficult to benchmark with other trusts”***(O)** (P53:Team Lead). These concerns contributed to a lack of confidence in acting upon audit feedback **(M)**. *“If this is replicated across the country*,* I’m not sure what conclusions you can draw from the report”* (P35:Team Lead-NC).

**Fig. 8:**
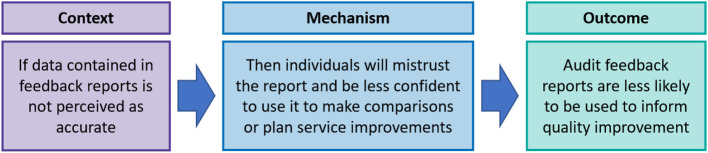
Refined IPT4 – Perceptions of data accuracy influence the use of feedback reports

When asked, 39% of all participants were aware of feedback being used to inform quality improvements within their organisation **(O)**. This included informing business cases for reviewing the skill mix of teams, funding for additional staff or resources. These findings suggest that reservations regarding data accuracy reduced confidence to use audit feedback for quality improvement. However, almost 40% of respondents were aware of data being used for quality improvement. The refined IPT4 is illustrated in Fig. 8.

## Discussion

This study used four proposed initial programme theories as a framework to investigate how and why community stakeholders participate in SSNAP. The aims of this study were to understand stakeholder perceptions and experiences of SSNAP in the community setting. These findings will inform adjustments in the audit intended to improve its efficacy as a quality improvement tool. Findings have been used to explore proposed theories, resulting in four refined initial programme theories.

Community stakeholders reported being engaged in the audit and described using feedback to inform a variety of quality improvements within their services. A number of challenges to audit participation were highlighted. These included the organisational culture, administrative support, online audit platform and ability of the audit to reflect the services delivered in this setting.

Individual perceptions of audit are informed by prior experiences. These include the organisational culture such as the behaviour of leaders or the response to feedback [36]. Participants in this study perceived organisations to be acute-focussed, resulting in a lack of leadership support for change in community services. This is in agreement with the wider audit literature that suggests that if change isn’t experienced in response to audit, this can fuel low motivation and disillusionment for clinical staff [37, 38]. An organisational culture of perceived leadership disinterest in audit impacts its ability to result in quality improvement. This study suggests these negative perceptions may be a potential barrier to future engagement with audit in the community.

Audit roles are rarely built into job specifications [39]. Instead, as highlighted by this study, audit activities are often perceived as an additional task assigned to clinicians rather than a resourced activity. Historically, community services have evolved to meet demand, and recruitment has prioritised clinical staff, resulting in a shortage of administrative support [40]. Consequently, community services in general often lack administrative support when compared to larger and more established acute hospitals. Absorbing administrative duties into clinical roles may be perceived as being a cost-effective use of limited resources in community services. However, this study highlights a lack of dedicated administrative support as a barrier to audit participation, impacting both audit efficacy and their clinical capacity. This echoes findings from Alvarado et al. where resources allocated to support participation in national clinical audit were reported as constraining its use as a tool for quality improvement [41].

This study highlighted challenges related to the online platform that contributed to data being incomplete. These concur with the wider literature where barriers such as duplicate data entry and incompatible IT systems are reported as barriers to audit participation [42]. Dixon-Woods et al., suggest that “*these mundane obstacles have a powerful impact on clinicians’ ability and willingness to complete data entry*” which in turn impacts audit participation [39]. Similarly to issues with administrative support, these factors may be more conspicuous in the community setting where services may lack the established infrastructure and centralised organisational resources found in acute services. These factors may contribute to the varying ability of audit to bring about improvements at different points in the stroke pathway, as identified by Cappadona et al. [3].

Perceived inconsistencies in audit practices between community teams were described as resulting in reduced confidence to use feedback to make comparisons. This concurs with Taylor et al., who found mistrust regarding auditing practises between hospital-based stroke teams prompted concerns regarding the use of audit data for commissioning purposes [43]. Both Wagner et al. and Sarkies et al. proposed that capturing the full scope of local workflows leads to greater clinician “buy-in” to the audit process [44, 45]. This is echoed by this study, where participants described a lack of confidence to engage with, or act upon audit findings that were perceived as failing to reflect the impact of community services. Further research is required to understand what measures stakeholders perceive would provide an accurate reflection of community services.

Despite reservations regarding data collected, respondents did describe using feedback reports to make comparisons between services. This suggests that in spite of the acknowledged limitations, stakeholders perceive there to be utility in data comparison. These findings are in agreement with the wider literature that suggests the use of routine data with known limitations is commonplace in healthcare [46]. Wolpert and Rutter coined the acronym FUPS to describe this flawed, uncertain, proximate and sparse data. Whereas FUPS data has previously been dismissed as unreliable, Wolpert and Rutter argue this data should be embraced. They propose the transparent reporting of FUPS, acknowledgment of limitations and triangulation with other findings in order to develop a greater understanding of complex health systems [47].

There has been a steady increase in the proportion of stroke survivors being discharged into community services in the UK over the last 10 years, reaching over 60% in 2023 [48]. This is partly a consequence of recent policy advances in terms of service specifications from NHS England [49] and the publication of evidence informed national clinical guidelines [50]. These initiatives reflect an increased emphasis on the efficacy and cost effectiveness of community-based services. Alongside this policy emphasis and evolution in the stroke pathway, there is a need for increased scrutiny of effectiveness and quality. If these policy initiatives are to be successful, consideration must be given to how best to evaluate delivery and outcomes both at a national and local level. The national stroke audit (SSNAP) offers an opportunity for such evaluation. However, the resources that community providers require to engage with and utilise their data must be considered.

This study has been conducted using realist methodology and as such is theory driven. Quantitative data provided contextual information such as the resources used, and activities undertaken as well as the perceived outcomes of the audit. Qualitative findings have expanded upon the proposed contextual features and illuminated potential mechanisms by which quality improvement may be achieved. The use of an online mixed methods survey is a novel methodological approach in RE, offering strengths and limitations as outlined below.

## Strengths and limitations

Broad representation was achieved from across both regions of England and categories of stakeholders. It is acknowledged that the self-selection of online surveys is inherently biased towards individuals with strong feelings regarding the subject matter [29]. This study used a self-selected sample of convenience and as such, the response rate as a proportion of the potential workforce was expected to be low. The anonymous nature of the survey, combined with an opportunity to expand using free text options, generated candid responses which may not have been the case in a face-to-face scenario. The distribution of role of participants reflects the reality of clinical practice, larger numbers of clinicians with fewer service managers and commissioners. However the smaller numbers of commissioners and senior managers did not support comparison between roles. Surveys lack the opportunity for probing or clarification. Therefore, these four refined initial programme theories will be taken forwards for further exploration. They will be used as a framework to explore the challenges identified in this study through realist interviews.

## Conclusion

The advancement of the evidence base and renewed policy emphasis on community rehabilitation necessitates an increased focus on performance and delivery of rehabilitation in this setting. Findings from this study highlight the work needed if the potential of the national stroke audit programme as a tool for quality improvement in the community rehabilitation is to match that seen in the acute sector. This is reliant on organisational support for audit, including leadership interest and the acknowledgement of audit as resourced and integral part of a service’s activity. In addition, the national audit must accurately reflect the stroke rehabilitation being delivered if services are to trust feedback and be confident in using it as a quality improvement tool in community stroke rehabilitation.

## Supplementary Information

Supplementary Material 1

Supplementary Material 2

## Data Availability

The datasets used and analysed during the current study are available from the corresponding author on reasonable request.

## Abbreviations

CHERRIES: Checklist for Reporting Results of Internet E-Surveys
CMO: Context Mechanism Outcome
GRAMMS: Good Reporting of a Mixed Methods Study
HQIP: Healthcare Quality Improvement Partnership
IPT: Initial Programme Theory
MDT: Multidisciplinary Team
NHS: National Health Service
RE: Realist Evaluation
SSNAP: Sentinel Stroke National Audit Programme
UK: United Kingdom
