# Supplementary material for: How and why do community stakeholders participate in the national stroke audit in England? Findings from a mixed-method online survey
Source: BMC Health Serv Res. 2024 Nov 6;24:1358. doi: 10.1186/s12913-024-11653-1 (PMC11539491; doi:10.1186/s12913-024-11653-1)
Supplement: Supplementary file 1 — Supplementary Material 1 [file 12913_2024_11653_MOESM1_ESM.docx]

**Checklist for Reporting Results of Internet E-Surveys (CHERRIES)**

| **Category** | **Item** | **Details or section no.** |
| --- | --- | --- |
| **Design** | Describe target population, sample frame | See section 2 Methods: Sampling and data collection |
| **Processes** | Institutional approval | Ethical approval for this study was granted by the University of Nottingham Faculty of Medicine and Health Sciences Research Ethics Committee (FMHS 387-1021). |
|  | Informed consent | See section 2 Methods: Sampling and data collection |
|  | Data protection | No personal information collected |
| **Development** | Development and testing | See section 2 Methods: Survey design |
| **Recruitment** | Open or closed | Open |
|  | Mode of contact | See section 2 Methods: Sampling and data collection |
|  | Advertising | See section 2 Methods: Sampling and data collection |
| **Administration** | Web or email | Automatic capture from Jisc Online™ web platform |
|  | Context | Potential participants directed to specific survey portal |
|  | Mandatory / voluntary | Voluntary survey, however all questions mandated for submission |
|  | Incentives | No incentives offered |
|  | Date / Time | December 1st 2021 to April 1st 2022 |
|  | Randomisation | No randomisation, all items presented in standard order |
|  | Adaptive questioning | Yes. If participants reported receiving audit feedback (item 14) 4 additional items were displayed.  If participants selected “other” from categorical responses (items 6, 10, 13a, 14a, 15a & 16a) free text responses were mandated. |
|  | Number of items | 18 items (first item participant consent)  & 7 sub items |
|  | Number of screens | 5 potential pages of survey items |
|  | Completeness check | All items mandated |
|  | Review step | Participants able to use back / forwards buttons to review and update responses prior to submission. |
| **Response rate** | Unique visitor site | Unique visitor numbers not recorded |
|  | View rate | 3300 views in total (may include duplicate views by same individual) |
|  | Participation rate | There were 3004 visits to 1^st^ page alone. An additional 86 progressed to page 2, and 4 to the final page without submitting a completed survey. 206 submitted completed surveys. |
|  | Completion rate | 206 / (86+4+206) x 100 = 70% |
| **Multiple entries** | Cookies | Not used |
|  | IP check | Not used |
|  | Log file analysis | Not used |
|  | Registration | Not used |
| **Analysis** | Handling of incomplete surveys | Not applicable – all questions mandated |
|  | Atypical timestamps | Not used |
|  | Statistical correction | No adjustments or weighting of scores |

Eysenbach, G., 2004. Improving the quality of Web surveys: Checklist for Reporting Results of Internet E-Surveys (CHERRIES). *Journal of medical Internet research*, *6*(3), p.e34.
