# Supplementary material for: How and why do community stakeholders participate in the national stroke audit in England? Findings from a mixed-method online survey
Source: BMC Health Serv Res. 2024 Nov 6;24:1358. doi: 10.1186/s12913-024-11653-1 (PMC11539491; doi:10.1186/s12913-024-11653-1)
Supplement: Supplementary file 2 — Supplementary Material 2 [file 12913_2024_11653_MOESM2_ESM.docx]

**Online Survey**

| **Item** | **Topic** | **Response option(s)** |
| --- | --- | --- |
| 1 | Participant consent |  |
| 2 | Role ^C^ | **Single response option:**   - Administrative Support - Rehabilitation Support Worker - MDT member > Band 5 - Team Lead (clinical) - Team Lead (non-clinical) - Service Manager - General / Divisional Manager - Commissioning |
| 3 | Region | **Single response option:** From a list of 20 regions in England |
| 4 | Understanding of audit process ^L^ | **5-point Likert scale*:**   - I understand the purpose of the audit - I am aware of evidence that performance is measured against - I understand what data is collected - I understand how data is collected locally - I understand how data is collected nationally - I understand how to access audit reports - I understand how to interpret audit reports - I understand how to use audit to inform service delivery - I understand how to share learning from the audit - I understand how to embed audit into routine practice |
| 5 | Understanding of role within audit ^C&L^ | **5-point Likert scale*:**   - I understand what my role is in the audit - I understand what activities I need to complete for the audit - I understand how to complete the required audit activities - I understand where to seek support with the audit |
| 6 | Audit activities undertaken ^C&L^ | **Multiple response options:**  From a list of 11 activities including “*Other*” e.g. data collection  Indicating “*Othe*r” triggers free text response box & prompt for details |
| 7 | Resource availability ^C&L^ | **5-point Likert scale*:**   - I have had sufficient raining for my role - I have had the equipment I need e.g. computers - I enough time in my working day to complete audit activities - I am able to prioritise audit activities when needed |
| 8 | Audit participation | **Yes / No:**  Are you able to fully complete the audit activities required for your role?  Selecting “*No*” triggers free text response box & prompt for details |
| 9 | Perceived benefits of participation ^C&L^ | **5-point Likert scale*:**   - Participating in audit benefits my service - Participating in audit benefits stroke patients in the community - Participating in audit benefits me personally - Participating in audit is a worthwhile use of my time |
| 10 | Resources used to support participation ^C^ | **Multiple response options:**  From a list of 13 resources including “*Other*” e.g. SSNAP website  Indicating “*Othe*r” triggers free text response box & prompt for details |
| 11 | Perceptions of data collected ^C^ | **5-point Likert scale*:**   - Data is complete for all stroke patients seen by the service - Data accurately reflects the rehabilitation delivered by this service |
| 12 | Metrics | **Yes / No:**  Is there any additional information you feel SSNAP should be collecting?  Selecting “Yes” triggers free text response box & prompt for details |
| 13 | Sharing of audit feedback report | **Yes / No / Don’t know:**  Is any information from the audit feedback report shared?  If “No” selected, skip to item 14. |
|  | a. In what situations? ^C^ | **Multiple response options:**  From a list of 8 situations including “O*ther*” e.g. team meetings  Indicating “*Othe*r” triggers free text response box & prompt for details |
| 14 | Participant experiences of audit feedback | **Yes / No:**  Do you receive or access audit feedback?  If “*No*” selected, skip to Item 15. |
|  | a. Main source of audit feedback ^C^ | **Single response option:**  From a list of 9 potential sources including “Other” e.g. Team Lead  Indicating “*Othe*r” triggers free text response box & prompt for details |
|  | b. Interpreting audit feedback report ^C&L^ | **5-point Likert scale*:**   - I am confident in interpreting the report - I have the skills to interpret the report |
|  | c. Feedback frequency | **Single response option:**  From 4 options including *“I don’t know”* |
|  | d. Is frequency adequate? | **Single response option:**   - Yes, about right - No, too often - No, not often enough |
|  | e. Audit feedback report accuracy ^C^ | **5-point Likert scale*:**   - I believe the report accurately reflects changes made by patients - I believe the report accurately reflects the rehabilitation delivered by my service - I believe the national report accurately reflects the rehabilitation delivered by other services - I believe the national report is trustworthy |
| 15 | Use of audit feedback report:  Comparison | **Yes / No / Don’t know:**  Is SSNAP feedback used to make comparisons? (By anyone in your organisation)  If “No” selected, skip to Item 16. |
|  | a. Purpose of comparison ^C&L^ | **Multiple response options:**  From a list of 6 situations including “O*ther*” e.g. Benchmarking  Indicating “*Othe*r” triggers free text response box & prompt for details |
| 16 | Use of audit feedback report:  Service improvement | **Yes / No / Don’t know:**  Is SSNAP feedback used in the planning of service improvement? (By anyone in your organisation)  If “No” selected, skip to Item 17. |
|  | a. Purpose of service improvement ^C&L^ | **Multiple response options:**  From a list of 5 situations including “O*ther*” e.g. funding for additional staff  Indicating “*Othe*r” triggers free text response box & prompt for details |
| 17 | Any additional uses for audit feedback not mentioned already? | **Yes / No:**  Are there any additional quality improvement uses for feedback you are aware of? (Not already reported)  Selecting “Yes” triggers free text response box & prompt for details |
| 18 | Any additional comments | Free text only |

^L^ Response options informed by literature

^C^ Response options informed by collaborator discussion

*Response options for 5-point Likert scale: *agree completely, agree partially, neither agree or disagree, disagree partially* and *disagree completely*
